# Reservoir cells no longer detectable after a heterologous SHIV challenge with the synthetic HIV-1 Tat Oyi vaccine

**DOI:** 10.1186/1742-4690-3-8

**Published:** 2006-01-27

**Authors:** Jennifer D Watkins, Sophie Lancelot, Grant R Campbell, Didier Esquieu, Jean de Mareuil, Sandrine Opi, Sylvie Annappa, Jean-Pierre Salles, Erwann P Loret

**Affiliations:** 1 UMR Univ. Med./CNRS FRE 2737, Faculté de Pharmacie, Université de la Méditerranée, 27 Bd Jean Moulin, 13385 Marseille, France; 2 SynProsis, Hôtel Technologique BP 100, Technopôle de Château Gombert, 13013 Marseille, France; 3 Department of Pediatrics, Division of Infectious Diseases, University of California San Diego, 9500 Gilman Drive, La Jolla, California 92093-0672, USA; 4 Laboratory of Molecular Microbiology, NIAID, National Institutes of Health, Bethesda, Maryland 20892-0460, USA

## Abstract

**Background:**

Extra-cellular roles of Tat might be the main cause of maintenance of HIV-1 infected CD4 T cells or reservoir cells. We developed a synthetic vaccine based on a Tat variant of 101 residues called Tat Oyi, which was identified in HIV infected patients in Africa who did not progress to AIDS. We compared, using rabbits, different adjuvants authorized for human use to test on ELISA the recognition of Tat variants from the five main HIV-1 subtypes. A formulation was tested on macaques followed by a SHIV challenge with a European strain.

**Results:**

Tat Oyi with Montanide or Calcium Phosphate gave rabbit sera able to recognize all Tat variants. Five on seven Tat Oyi vaccinated macaques showed a better control of viremia compared to control macaques and an increase of CD8 T cells was observed only on Tat Oyi vaccinated macaques. Reservoir cells were not detectable at 56 days post-challenge in all Tat Oyi vaccinated macaques but not in the controls.

**Conclusion:**

The Tat Oyi vaccine should be efficient worldwide. No toxicity was observed on rabbits and macaques. We show *in vivo *that antibodies against Tat could restore the cellular immunity and make it possible the elimination of reservoir cells.

## Background

The HIV-1 Tat protein plays important roles in the virus life cycle and maintenance of HIV-1 infected CD4+ T cells [1,2]. It is a *trans*-activating regulatory protein that stimulates efficient transcription of the viral genome, which requires structural changes of Tat to bind to a RNA stem-loop structure called TAR [3,4]. However, Tat differs from other HIV-1 regulatory proteins because it is rapidly secreted by CD4^+ ^T cells following HIV-1 infection, and extra-cellular Tat is suspected to be directly involved in the collapse of the cellular immune response against HIV-infected cells [2] and directly contributes to the pathology of AIDS [5]. Extra-cellular Tat inhibits macrophage responses by binding to the Fas ligand membrane receptor [6] and inhibits cytotoxic T cell (CTL) responses due to its ability to cross cell membranes and induce apoptosis of uninfected T cells [7,8] via interaction with tubulin [8-10]. In addition, a number of studies have shown that the presence of antibodies against Tat blocks the replication of HIV-1 *in vitro *and is related to non-progression to AIDS [11-13]. Moreover, it has been shown that a HIV-1 Tat-specific cytotoxic T lymphocyte response is inversely correlated with rapid progression to AIDS [14]. Further studies have emphasized the hypothesis that anti-Tat CTLs are important in controlling virus replication early after primary infection [14,15].

The discovery of the extra-cellular functions of Tat in the inhibition of the cellular immune response against HIV-infected cells constitute the rationale to develop a vaccine against HIV targeting Tat [16]. However, the development of a Tat vaccine may face the same problems encountered with HIV-1 envelope proteins as Tat exists in different sizes (86 to 101 residues) and mutations exist that induce structural heterogeneity [17]. The 2D NMR studies of two active Tat variants from Europe and Africa confirmed this structural heterogeneity, although a similar folding appears to exist among Tat variants [18-20]. Currently, there are five main HIV-1 subtypes in the world: subtypes A (25 %) and C (50 %) are predominant and are found mainly in Africa, India and South America; subtype B (12 %) is found mainly in Europe and North-America; subtype D (6%) is found in Africa and subtype E (4 %)(a recombinant form known as CRF_01AE), is found mainly in South East Asia [21]. Tat variability follows this geographical diversity with mutations of up to 38 % observed among Tat variants from A, B, C, D and E HIV-1 subtypes that do not alter Tat functions but do not allow cross recognition with Tat antibodies [22].

Up to now, the two main vaccine strategies against Tat use a recombinant protein corresponding to a short 86 residue version of a subtype-B European Tat variant that is either inactivated [11] or has full activity [23]. These two approaches were tested on macaques followed by a homologous SHIV challenge [24,25]. A significant decrease of viremia was observed in these two studies carried out respectively on Cynomolgus [24] and Rhesus macaques [25], without showing complete protection during primary infection. A recent study showed long term control of infection following homologous SHIV challenge on Tat-vaccinated Cynomolgus macaques [26]. However, immunization with a subtype B Tat variant of 86 residues does not stimulate an efficient response against subtype A and C Tat variants [27]. Moreover, most Tat variants found in the field are of 101 residues [4].

Over the last 20 years, several HIV vaccine studies have been tested using a homologous SHIV/macaque model and some have met with success [28]. However, these were not followed by success in clinical trials [29], possibly due to the high genetic diversity of HIV-1. This is why heterologous SHIV challenge in macaques, using a genetically distinct virus, is now recommended to determine if a vaccine can be effective against HIV-1 infection in humans and corresponds to the most significant *in vivo *experiment after clinical trials [28].

The interest to develop a Tat vaccine rose with the discovery that seropositive long-term non-Progressor (LTNP) patients had a higher level of Tat antibodies than seropositive Rapid Progressor (RP) patients [13]. However, LTNP patients are unable to eradicate HIV since they still have HIV released from reservoir cells. Another category of patients, the highly exposed persistently seronegative (HEPS), appears to be more interesting since they were in contact with the virus, they have developed a strong cytotoxic T lymphocyte (CTL) response against viral proteins and have retro converted to become seronegative [30]. There is a very low prevalence of HEPS among adults and it could be possible that the HEPS phenotype is due to innate immunity [31].

Although HEPS patients have normally no detectable virus, it was possible to isolate and clone a HIV-1 strain from patients in a cohort in Gabon [32] that could be now classified as HEPS. This strain called HIV-1 Oyi has genes similar to regular HIV-1 strains except the *tat *gene, which had mutations never found in other Tat variants [16]. The epidemiological survey was carried out on a sample of 750 pregnant women and 25 were identified as seropositive [32]. From these 25 seropositive women, 23 rapidly retro converted and became HEPS. All the HEPS women were infected with HIV-1 Oyi. The high proportion of HEPS phenotype in this cohort (92%) indicated that the retro conversion was probably due to an acquired immunity and not an innate immunity. Ten years after the publication of this epidemiological survey, the 23 women were in good health and the HIV was no longer detectable in their blood [22]. Immunization with Tat Oyi raises antibodies in rabbits that are able to recognize different Tat variants even with mutations of up to 38 %, which is not possible with other Tat variants [22]. Tat Oyi appears to induce a humoral immune response against three-dimensional epitopes that are conserved in Tat variants in spite of 38% mutations [22]. Moreover, Tat Oyi has a similar structure to active Tat but is unable to *trans-*activate [20].

This study is the first step of pre-clinical studies of a vaccine using a synthetic protein of 101 residues. Synthetic vaccines are developed for many years because they could be safer regarding biological vaccines, i e vaccines made from inactivated pathogens or recombinant proteins. However, most of the vaccines commercially available up to now have a biological origin. Very few synthetic vaccines were able to demonstrate their efficacy *in vivo *against a pathogen such as bacteria or virus due to the short size of the peptides that can constitute only linear epitopes, while 3D epitopes are the most susceptible to trigger an immune response that neutralize a pathogen. This is why, one of the objective of this study was to determine a vaccine formulation suitable for human use to prepare clinical trials, as a previous study with Tat Oyi was carried out using complete Freund adjuvant [22]. We evaluated the antibody responses raised in rabbits by Tat Oyi complemented with adjuvants authorized for human use and we determined formulations providing similar results previously obtained with the Freund adjuvant [22]. Vaccination with Tat Oyi on seven Rhesus macaques provided an excellent model to test *in vivo *the efficacy of this synthetic vaccine before clinical trials. Furthermore, the vaccinated macaques were challenged with a European SHIV. This was a heterologous SHIV challenge and no success in heterologous SHIV were published until now.

## Results and discussion

We selected four adjuvants (Calcium phosphate, Montanide, Adju-Phos and Alhydrogel) to develop different vaccine formulations with our synthetic protein Tat Oyi. The usual dose of aluminium for human vaccines is around 0.5 mg [33] and at this concentration, approximately 90 % of 100 μg of Tat Oyi adsorbed to both aluminium containing adjuvants (Adju-Phos and Alhydrogel). For these two reasons, we decided to carry out our inoculations at 0.5 mg Al per dose of vaccine for both Adju-Phos and Alhydrogel. For the calcium phosphate gel, we achieved 92 % adsorption using 1 mg Ca per 500 μl dose while only 62% adsorption using 0.5 mg Ca in the same volume. Montanide adjuvant (70 %) was used because it is a metabolizable oil that can be used for human vaccination and has chemical properties similar to those found in the Freund adjuvant as used in our first vaccination studies [22].

Twelve rabbits were immunized with the four formulations (three rabbits for each formulation) and we analyzed the antibody responses against five Tat variants representative of subtypes A, B, C, D, and E (Table I). No antibody response was observed using the calcium phosphate gel and the aluminium phosphate adjuvants at 60 days post-inoculation. However, at 90 days post-inoculation, a strong antibody response was observed using these two adjuvants against five Tat variants (Table I). The best humoral response against Tat oyi was obtained using Montanide ISA720 (titer: 128,000 against Tat Oyi) at both 60 and 90 days post-inoculation. However, Montanide ISA720 and Calcium phosphate appear to be the most suitable adjuvants to complement the synthetic protein Tat Oyi, due to the absence of toxicity and the heterologuous immunity compared with different Tat variants observed after vaccination (Table I).

**Table I:** Titre of pooled rabbit sera against different Tat variants (60 and 90 days post-inoculation)

	Montanide ISA720 J60			Montanide ISA720 J90			Preimmune		
	titre	mean	SD	titre	mean	SD	titre	mean	SD

Oyi	128,000	6,700E-02	1,500E-03	128,000	7,000E-02	2,646E-03	320	6,733E-02	2,082E-03
Ug11RP	16,000	6,867E-02	1,443E-03	16,000	6,867E-02	2,309E-03	160	6,867E-02	1,155E-03
Eli	32,000	7,017E-02	1,041E-03	64,000	7,000E-02	1,000E-03	160	7,200E-02	2,000E-03
96Bw	8,000	7,400E-02	3,279E-03	16,000	6,933E-02	5,774E-04	320	8,000E-02	6,000E-03
CM240	32,000	6,683E-02	5,774E-04	1,000	6,600E-02	1,732E-03	320	6,767E-02	1,155E-03
HXB2	64,000	6,233E-02	1,041E-03	16,000	6,844E-02	2,143E-03	160	6,033E-02	2,887E-03

	Aluminium Hydroxide J60			Aluminium Hydroxide J90			Preimmune		

	titre	mean	SD	titre	mean	SD	titre	mean	SD

Oyi	64,000	6,700E-02	8,660E-04	16,000	6,767E-02	2,082E-03	80	6,700E-02	8,660E-04
Ug11RP	16,000	6,850E-02	5,000E-04	2,000	6,700E-02	9,313E-10	160	6,850E-02	5,000E-04
Eli	64,000	6,550E-02	9,313E-10	8,000	6,533E-02	5,774E-04	160	6,550E-02	9,313E-10
96Bw	32,000	7,167E-02	2,887E-04	1,000	6,733E-02	1,528E-03	160	7,167E-02	2,887E-04
CM240	32,000	6,967E-02	7,638E-04	1,000	6,633E-02	5,774E-04	80	6,967E-02	7,638E-04
HXB2	64,000	6,650E-02	1,000E-03	16,000	6,500E-02	1,000E-03	160	6,650E-02	1,000E-03

	Calcium Phosphate Gel J90			Preimmune					

	titre	mean	SD	titre	mean	SD			

Oyi	32,000	8,033E-02	1,528E-03	160	6,700E-02	2,887E-03			
Ug11RP	16,000	6,750E-02	2,517E-03	320	6,733E-02	5,774E-04			
Eli	32,000	7,975E-02	3,786E-03	160	7,567E-02	1,155E-03			
96Bw	8,000	7,000E-02	2,000E-03	320	6,567E-02	5,774E-04			
CM240	16,000	7,150E-02	3,215E-03	320	6,733E-02	1,528E-03			
HXB2	128,000	6,600E-02	3,606E-03	80	6,533E-02	5,774E-04			

	Aluminium Phosphate J90			Preimmune					

	titre	mean	SD	titre	mean	SD			

Oyi	32,000	6,900E-02	2,082E-03	320	6,800E-02	1,000E-03			
Ug11RP	16,000	6,800E-02	2,082E-03	80	6,700E-02	1,414E-03			
Eli	32,000	6,875E-02	2,646E-03	320	7,067E-02	1,528E-03			
96Bw	8,000	6,875E-02	2,309E-03	160	7,367E-02	3,215E-03			
CM240	16,000	7,075E-02	1,528E-03	160	7,433E-02	1,155E-03			
HXB2	32,000	6,825E-02	2,309E-03	160	7,100E-02	2,646E-03			

Titre corresponds to the reciprocal of the last positive dilution obtained by ELISA (cut-off : mean of preimmune sera + 3 S.D.)

**Figure 1:**
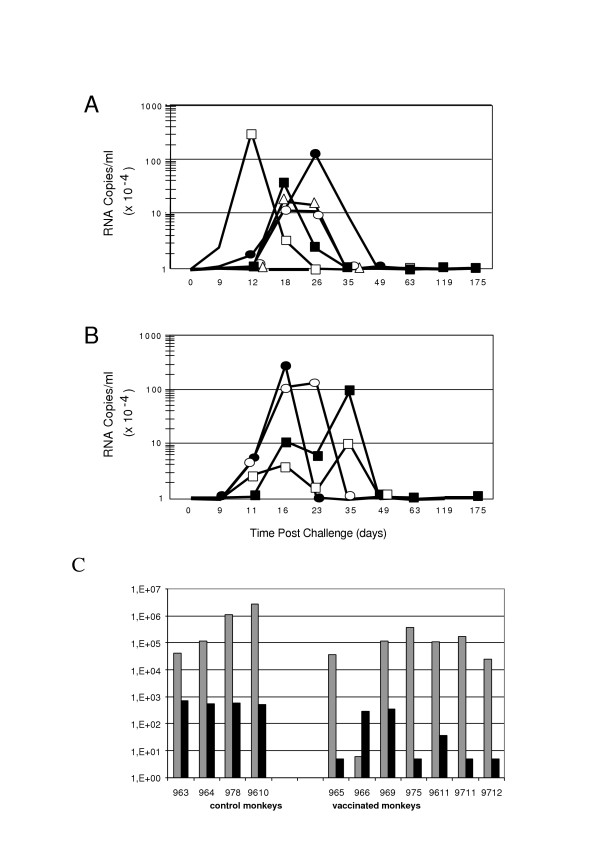
Viral load of rhesus macaques vaccinated with Tat Oyi (**panel A**) and control macaques vaccinated with β-gal (**panel B**) following SHIV challenge (SHIV-BX08). The 965 (white square), 966 (no symbol), 969 (black circle), 975 (black square), 9611 (white circle), 9711 (white triangle) and 9712 (black triangle) macaques are the Tat Oyi vaccinated macaques. The 963 (white square), 964 (black square), 978 (white circle) and 9610 (black circle) Macaques are the controls vaccinated with β-gal. Two vaccinated macaques (965 and 969) on five had a viremia up to or superior to 1 millions RNA copies/ml that similar to controls. Macaque 966 had a viremia almost undetectable after the first SHIV challenge and remained at the same level in spite of a second challenge with SHIV 162P 3.2 seven weeks after the first challenge. The other macaques were not challenged twice. Control macaque 963 had an unexpected low viremia. **Panel C**: Grey bars indicate the post infection viremia in the plasma at two weeks and the black bars indicate viremia at nine weeks post-infection of the challenged macaques. Macaque 966 has a higher viremia at nine weeks due to its second SHIV challenge.

**Figure 2:**
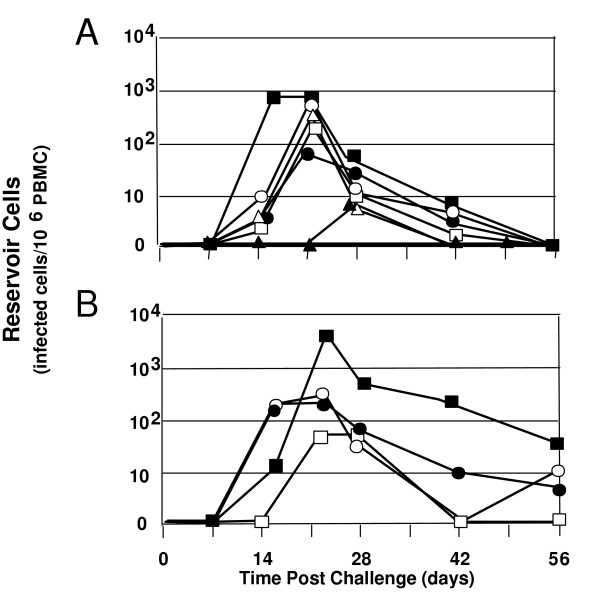
HIV infected CD4 T cell (reservoir cells) in rhesus macaques vaccinated with Tat Oyi (**panel A**) and control macaques vaccinated with β-gal (**panel B**) following SHIV challenge. The 965 (white square), 966 (no symbol), 969 (black circle), 975 (black square), 9611 (white circle), 9711 (white triangle) and 9712 (black triangle) Macaques are the Tat Oyi vaccinated Macaques. The 963 (white square), 964 (black square), 978 (white circle) and 9610 (black circle) Macaques are the controls vaccinated with β-gal. The upper panel shows that no reservoir cells were detectable in the seven Tat Oyi vaccinated macaques after 56 days although macaques 965 and 969 had high viremia peaks (Fig 1). Interestingly, no reservoir cells were detectable at any time for macaque 966 even after its second SHIV challenge.

A heterologous SHIV-BX08 challenge carried out on seven macaques vaccinated with Tat Oyi/Montanide ISA720 and four control macaques vaccinated with β-galactosidase that were used also as control for another vaccine trial [34]. Figure 1 shows the viremia as revealed by SHIV RNA copy number in the sera of macaques after SHIV challenge. Similarly to what is observed in human a couple of months after HIV infection, both Tat Oyi vaccinated macaques and controls had an undetectable viremia 63 days after the SHIV challenge (Fig 1). In addition, virus isolation and cytoviremia was measured by co-cultivation of PBMC's with non-infected human cells at the day of challenge and each week afterwards and allow to estimate the level of reservoir cells (Fig 2). Five on seven Tat Oyi vaccinated macaques showed a better control of viremia compared to control macaques (Fig 1). Reservoir cells were not detectable at 56 days post-challenge in all Tat Oyi vaccinated macaques but not in the controls (Fig. 2).

**Figure 3:**
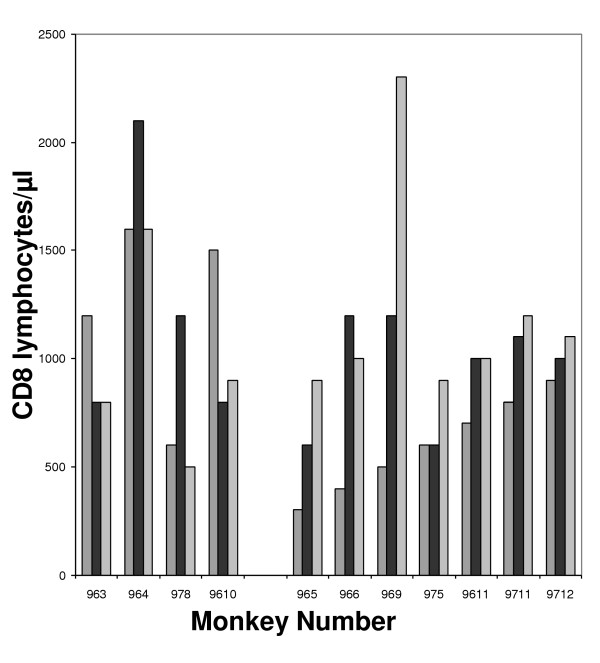
CD8+ cell count of challenged Macaques. The 963, 964, 978 and 9610 Macaques are the controls. The 965, 966, 969, 975, 9611, 9711 and 9712 Macaques are the vaccinated Macaques. Striped histograms represent the CD8+ cell count at the day of challenge. Black histograms represent the CD8+ cell count 9 weeks post-challenge whilst grey histograms represent the CD8+ cell count 18 weeks post-challenge.

It has been shown in SHIV challenge that plasma viremia in the first peak does not correlate with survival whereas plasma viremia levels of the second peak at or about six weeks post-infection were highly predictive of relative survival [35]. In our vaccine trial, panel C in figure 1 shows that plasma viral RNA levels were significantly lower in the vaccinated Macaques compared to the controls at nine weeks post-infection (p = 0.009 using Mann-Whitney test). While we did not observe major differences in the level of CD4 cells between vaccinated and non vaccinated macaques (data not shown), we did observe an augmentation of the number of CD8 lymphocytes in Tat Oyi vaccinated macaques (Fig. 3). However, we did not determine if these CD8 are HIV specific CTL. It is interesting to observe that before the SHIV challenge, control macaques had a higher level of CD8 compared to Tat Oyi vaccinated macaques. Control macaques were immunized with the Semliki Forest Virus (SFV) *lac Z *expressing β-galactosidase that boost the CD8 response [34]. This high level of CD8 were not HIV specific in control macaques and they had no antibodies against Tat. Therefore, we think that the decreased level of CD8+ cells in control macaques after the SHIV challenge could be due to extracellular Tat, since the SHIV infection should have increased the CD8 response as observed for SFV.

**Figure 4:**
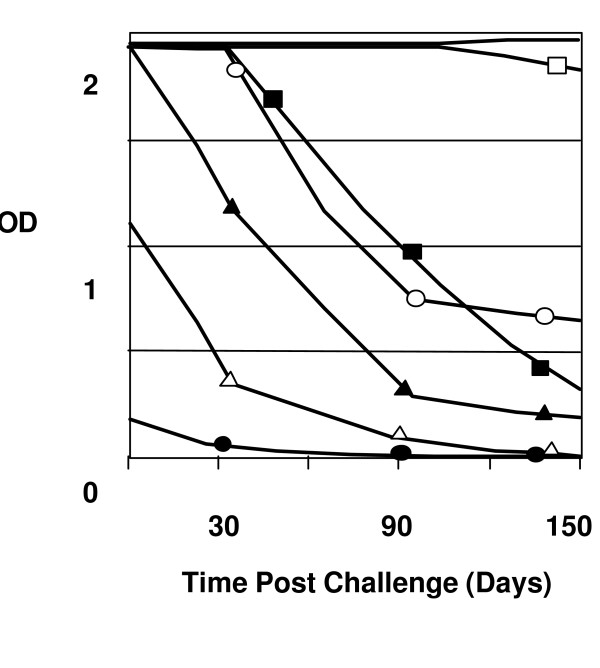
Antibody response against Tat for the seven macaques vaccinated with Tat Oyi. The 965 (white square), 966 (no symbol), 969 (black circle), 975 (black square), 9611 (white circle), 9711 (white triangle) and 9712 (black triangle) Macaques are the Tat Oyi vaccinated Macaques. Macaque 966 in the top had the best response against Tat and turned to have the best control of the viremia with no reservoir cells detected (Fig 1 & 2). The left axis shows the OD of 1/100 sera dilution.

**Figure 5:**
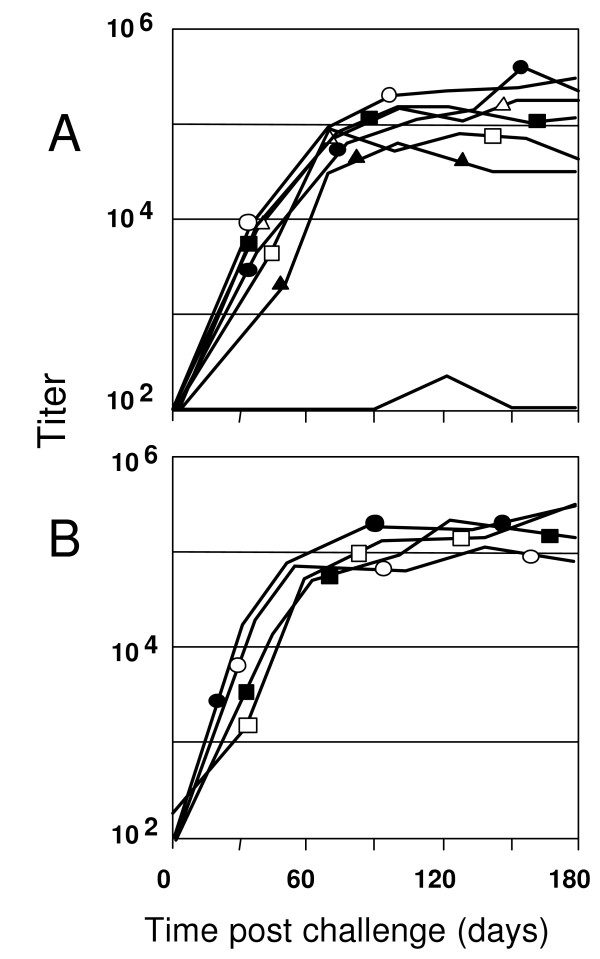
Antibodies titers against GP120. The 965 (white square), 966 (no symbol), 969 (black circle), 975 (black square), 9611 (white circle), 9711 (white triangle) and 9712 (black triangle) Macaques are the Tat Oyi vaccinated Macaques. The 963 (white square), 964 (black square), 978 (white circle) and 9610 (black circle) Macaques are the controls vaccinated with β-gal. Six from the seven macaques vaccinated with Tat Oyi had a high level of GP120 antibodies (panel A) similar to the macaques controls (panel B). Antibodies against GP120 appears to not have play a role in the elimination of reservoir cells. This is well illustrated with macaque 966 (Panel A) that had no antibody against GP120 after the first challenge SHIV and a low level of antibodies after its second SHIV challenge.

All Tat-vaccinated macaques, with the exception of Macaque 969, developed a strong anti-Tat antibody response (Fig 4), which correlated with an efficient reduction in viremia at nine weeks post-infection (Fig 1C). This was best demonstrated by monkey 965, which had a strong anti-Tat antibody titer and a significantly reduced viremia nine weeks post-infection despite a high viremia in the primary phase (Fig 1C). To a lesser extent, macaque 9711 shows the same relationship between the level of anti-Tat antibody and the viremia at nine weeks (Fig 1C). Moreover, the control of viremia in Tat Oyi vaccinated macaques was not due to antibodies raised against the HIV envelope proteins since the four SHIV challenged control macaques had high anti-gp120 antibody titers. Overall, gp120 antibody titres were similar in control and Tat Oyi vaccinated macaques (Fig 5).

Macaque 966 did react differently from the other Tat Oyi vaccinated macaques and is the most interesting. It was the one to have an almost complete immunity against SHIV BX08 with a viremia peak around 300 RNA copies per ml whilst most of the others macaques had viremia peaks between 100 000 and 3 000 000 RNA copies per ml (Fig 1). Interestingly, almost no antibodies against gp120 were detectable and no virus could be isolated from cultured PBMC's (Fig 2). To verify this strong immunity, macaque 966 was challenged a second time with another heterologous SHIV 162P 3.2 seven weeks after the SHIV BX08 challenge (Roger Legrand, Personal communication). This second challenge explains the higher viremia peak at nine weeks post-infection compared to the other Tat Oyi vaccinated macaques (Fig 1C), which rapidly decreased to an undetectable level. It is interesting also to note that antibodies against gp120 were observed with macaque 966 following the second SHIV challenge that also rapidly declined (Fig 5). Results observed with macaque 966 are very important and constitute the best proof of concept for the Tat Oyi vaccine and its rational as previously described [22]. Macaque 966 had the highest titer of anti-Tat antibody (Fig 4), the lowest viremia (Fig 2) and no detectable virus from cultured PBMCs (Fig 1). Macaque 965 had nearly identical level of anti Tat antibodies but was not able to control its viremia as macaque 966. It is possible that innate immunity helped macaque 966, but it is interesting to note that antibodies against gp120 disappeared rapidly for macaque 966 (Fig 5), similarly to what was observed with the patients infected by HIV-1 Oyi in Gabon [32] and HEPS patients [30].

Conflicting results appears in Tat vaccine studies in nonhuman primate viral challenges models ranging from no protection [34,36-38] to significant [39,24,25], long term protection [26]. Although these conflicting results could be explained by differences in immunization regimen, viral stock, route of viral challenge and animal species, the result of two studies using similar viral vector expressing Tat, Env and Gag and giving opposite conclusion is puzzling [36,39]. One study shows the efficacy of vectored Tat but not Gag and Env [39], while another study showed efficacy of vectored Gag and Env but not Tat [36]. These conflicting results could be due to a homologous challenge in the first study [39] and a heterologuous challenge in the second study, since the second study use the Tat Jr sequence instead of the homologuous Tat Bru sequence for the vaccine [36]. HIV-1 Jr and HIV-1 Bru are B subtypes but their Tat sequences have non conservative mutations inducing conformational changes [16]. The mutations between the vaccine and the challenge virus might explain the lack of efficacy of the Tat vectored vaccine in the second study [36]. Of course, the second study more closely resembled reality since a vaccinated person will not likely be exposed a homologous virus infection. It is possible that the study by Silvera *et al*. would have had an different outcome had heterologous *gag *and *env *genes been used in the SHIV challenge [36]. These studies outline how mutations can affect Tat cross recognition as shown in former studies [22,27].

## Conclusion

Three adjuvants authorized for human use trigger an immune response with Tat Oyi similar to what was observed with the complete Freund adjuvant in a former study [22]. No local or systemic toxicity or adverse effects were observed in rabbits and macaques with vaccine doses superior to those planed for clinical trials. Furthermore, the synthetic protein Tat Oyi is pharmacologically stable in solution for at least a period of one month, which is a requirement for mass vaccination (data not shown). Although a low viremia was not achieved for all macaques, reservoir cells were no longer detectable 56 days after a heterologuous challenge. Taken together, these results suggest that a Tat Oyi synthetic protein could be an excellent component of a vaccine targeting HIV-1 and could provide an appropriate treatment against HIV-1 in both developing and industrial countries. On a fundamental point of view, the decreased level of CD8 cells in the control macaques suggests an important role of extra cellular Tat in the immunodeficiency induced by the HIV-1. We hope to be able to confirm in phase I/II clinical trial with seropositive patients that a therapeutical effect can be obtained from the Tat Oyi vaccination. This therapeutic effect might result, firstly, in a reduced viremia and stable CD4 cells level following an interruption of the antiretroviral treatment. We believe this vaccine will not prevent sero negative people from HIV infection, however it could avoid the collapse of the cellular immunity, and therefore a therapeutic effect could be expected with the eradication of the virus titres and viral reservoir as is observed with HEPS patients. This vaccine could be also the only affordable therapy for millions of seropositive patients that have no access to antiretroviral treatment.

## Methods

### Tat variants and adjuvant formulations

Tat variants were assembled in solid phase synthesis with an ABI 433A peptide synthesizer with FASTMoc chemistry according to the method of Barany and Merrifield [40] as previously described [20,41]. The calcium Phosphate gel adjuvant was obtained from Brenntag Biosector (Denmark). The adjuvant based on a metabolizable oil with a mannide mono-oleate emulsifier called Montanide ISA720 was obtained from SEPPIC Ltd (Paris, France). The two aluminum-containing adjuvants, aluminum hydroxide (Alhydrogel 2 %, Superfos Biosector a/s,) and aluminum phosphate (Adju-Phos, Superfos Biosector a/s), were kindly provided by Vedbaeck (Denmark). Experiments were conducted to assess the presence of soluble antigen in the supernatant liquid of adsorbed experimental vaccines. Tat Oyi was added to the gel and gently shaken for 24 h at room temperature. Samples were centrifuged at 313 g for 15 min at room temperature. Supernatant was aspirated and protein concentration was determined using Bradford reagent. Protein adsorption by aluminum-containing adjuvants was studied in 500 μl suspensions containing a quantity of adjuvant equivalent to 0.7, 0.5 or 0.3 mg Al.

### Immunization protocols for rabbits and macaques

Twelve specific pathogen-free New Zealand rabbits (Elevage Scientifique des Dombes, Romans, France) were immunized with 100 μg of Tat Oyi and four different formulations (three rabbits for each formulation): aluminum hydroxide (0.5 mg of Al) in phosphate buffer 20 mM pH 6.5; aluminum phosphate (0.5 mg of Al) in sodium acetate buffer 20 mM pH 6.5; calcium phosphate gel (1 mg of Ca) in phosphate buffer 20 mM pH 7; and Montanide ISA720 (70%) in phosphate buffer 20 mM pH 6.5. Each rabbit was boosted three times at 20, 40 and 75 days after the first immunization. Sera were collected before immunization, and then 60 and 90 days after the first immunization. No death or injuries were observed during or as a consequence of the immunization for the full time of the experiment. The study on Macaques included eleven rhesus macaques of Chinese origin. These macaques were housed at the Primate Research Center at Rennemoulins (Institut Pasteur, France) and handled under ketamine hydrochloride anesthesia (Rhone-Mérieux, Lyon, France) according to European guidelines for animal care (Journal Officiel des Communautés Européennes, L358, 18 décembre 1986). The animals were checked to be virus-isolation negative, as well as sero-negative for SIV and simian retrovirus type D before entering the study. Seven macaques were immunized subcutaneously with Tat Oyi (100 μg) and the adjuvant Montanide ISA 720. Boosts were given at 1, 2 and 3 months after the first immunization. The control was four macaques immunized with the Semliki Forest Virus *lac Z *expressing β-galactosidase [34]. No death or injuries were observed during or as a consequence of the immunization for the full time of the experiment.

### SHIV challenge

The seven macaques vaccinated with Tat Oyi were included in a SHIV challenge assay called RIVAC sponsored by the ANRS. The purpose of the RIVAC assay was to compare ten vaccine approaches on five to seven macaques with the same SHIV challenge model. Only results obtained with three vaccine approaches have been published [34]. The challenge strain was SHIV-BX08, derived from SIVmac239 [34]. This is a hybrid virus expressing the gp120 subunit of the R5, clade B, primary HIV-1 isolate BX08 and the gp41 subunit of HIV-1 LAI [42]. The *tat *and *rev *genes are also from HIV-LAI, whereas the *gag*, *pol*, *vif*, *vpx *and *nef *genes are from SIVmac239. The animals were challenged intra-rectally (IR) seven months after the first immunization. The virus stock used for challenge was amplified on human PBMC and 10-fold serial dilutions where used for inoculation of rhesus macaques. The undiluted challenge dose contained 337 +/- 331 AID_50 _for IR administration, as determined by the method of Spouge [43]. Tat vaccinated and control animals were sedated with ketamine hydrochloride (10 mg/kg i.m.)

### Serological tests

ELISA were carried out as previously described [22] with a minor change. Maxisorp U96 immunoplates (Nunc) were coated with 100 μl of Tat Oyi diluted at 2,3 μg/ml in phosphate buffer 100 mM pH 6 overnight at 4°C. This experiment was repeated three times.

### HIV infected CD4 T cell or reservoir cell count

Reservoir cells counts was carried out with the cell-associated viral load method [44]. Virus isolation was carried out by co-cultivation of macaque PBMC with PHA-stimulated human (donor) PBMC. Viral RNA was extracted from 200 μl of plasma collected on EDTA using the High Pure RNA Kit from Roche (Mannheim, Germany) and stored frozen at -80°C. 10 μl of the extracted material were then submitted to reverse transcription and PCR for amplification as described previously [34].

### Cell count

Counting of CD4^+^, CD8^+^, CD3^+ ^and CD20^+ ^cells was performed as described previously [45].

### Statistical analysis

Statistical analysis of serological data was carried out using the Mann-Whitney test or one-way Anova test using Minitab Release 14. We considered that the difference between two samples was significant if the P-value was less than 0.05.

## List of abbreviations

HIV, human immunodeficiency virus

PBMC, Peripheral Blood Mononuclear Cell

Tat, Trans activator protein

## Competing interests

The author(s) declare that they have no competing interests.

## Authors' contributions

JDW carried out ELISA test on rabbits, interpreted the SHIV challenge's results and participated to the redaction of the manuscript. DE participated to ELISA test on rabbits. GC participated to ELISA test on rabbits and the redaction of the manuscript. SL, SO and JM participated in the immunization protocol of the preformulation's studies. DE, GC, SO, SA synthesized the proteins for rabbit ELISA. JPS interpreted SHIV challenge results and participated to the redaction of the manuscript. EPL immunized rabbits, synthesized and provided Tat Oyi for macaque immunization, and wrote the manuscript.
